# Thermally Primed *Zostera muelleri* Seeds Exhibit Higher Germination Rates Than Those From Ambient Conditions

**DOI:** 10.1002/ece3.70362

**Published:** 2024-10-02

**Authors:** Tom Moir, Megan J. Huggett, Timothy M. Smith, Troy F. Gaston

**Affiliations:** ^1^ School of Environmental and Life Sciences University of Newcastle Ourimbah New South Wales Australia; ^2^ Centre for Tropical Water & Aquatic Ecosystem Research James Cook University Cairns Queensland Australia

**Keywords:** climate change, estuary, seagrass, seed germination, thermal effects, Zostera

## Abstract

Seagrasses provide critical ecosystem services such as carbon sequestration, sediment stabilisation and nursery habitat for juvenile fish. *Zostera muelleri* is ubiquitous within Australian and New Zealand estuaries, however, as a species is relatively understudied. We sourced seeds from a thermally affected east Australian estuary and investigated whether germination rates differed between ambient and thermally affected seeds over a variety of temperatures (16°C–28°C) to determine how seagrass systems might react in a warming climate. Germination for the experiment was low and totalled 5% of all seeds; however, similar numbers are typical in seed germination studies. Germination was highest at 16°C and was enhanced through the simulation of a 48‐h freshwater pulse. Thermally affected sites germinated faster and had greater mean maximum germination when compared to control sites regardless of experimental temperature. These findings indicate that *Z. muelleri* in this system may be exhibiting transgenerational plasticity due to the thermal stress the parent experiences. This result provides an alternate viewpoint to the current literature by suggesting that unknown transgenerational effects may provide *Z. muelleri* with greater germination plasticity against temperatures expected under predicted climate change scenarios than previously expected.

## Graphical abstract

Climate change is threatening seagrass ecosystems globally; however, some species will have the capacity to adapt. Our results indicate that seeds sourced from adult plants residing in thermally affected areas germinate faster and more often than those sourced from ambient conditions.

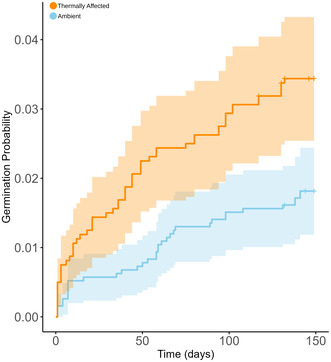

## 1 Introduction

Climate change threatens marine ecosystems globally and stands to disrupt thermal optimums to which species have adapted (IPCC, 2022). For seagrasses, a rise in temperature may see species disappear as temperature extremes in the shallow water rise (York et al. 2013). It is likely that projected increasing temperatures and salinities in estuaries (Scanes, Scanes, and Ross 2020) will significantly inhibit temperate seagrass species' ability to successfully germinate.

Seagrasses are a prominent habitat in estuaries on the east coast of Australia, which support fish as critical nursery habitat (Heck, Hays, and Orth 2003; Nagelkerken 2009; Olson et al. 2019). Further, they significantly contribute to sediment stabilization (Christianen et al. 2013), carbon sequestration (Macreadie et al. 2014; Macreadie, York, and Sherman 2014) and consequently have significant value economically (Costanza et al. 2014; Röhr et al. 2016). Seagrass habitats are threatened worldwide from a variety of factors such as urbanisation, pollution and climate change, which have resulted in a decline of 25% of all known seagrass meadows between 2000 and 2010 (Turschwell et al. 2021). However, seagrasses are highly resilient to significant short‐term changes in temperature, light and salinity associated with near‐shore ecosystems (York et al. 2013). A key component forming this resiliency in seagrass ecosystems has been attributed to efficient regenerative strategies employed through asexual or sexual reproduction (Smith et al. 2016). Australia hosts a rich variety of seagrasses on the east coast; however, *Zostera muelleri* is the most prominent with a widespread geographical distribution, forming large monospecific beds in estuaries along the east Australian coastline (York et al. 2013; Macreadie et al. 2018). Reproduction in *Z. muelleri* can be achieved through asexual or sexual methods with a combination of both providing optimal reproduction (Kendrick et al. 2012; Macreadie et al. 2014; Macreadie, York, and Sherman 2014; Stafford‐Bell, Chariton, and Robinson 2015). Asexual clonal growth through rhizome elongation allows efficient expansion within a meadow which aids in population resilience and can result in more successful colonization of bare substrates but limits genetic diversity (Kendrick et al. 2012; Macreadie et al. 2014; Macreadie, York, and Sherman 2014; Stafford‐Bell, Chariton, and Robinson 2015). Sexual reproduction allows for more genetic diversity, and detached reproductive shoots can travel long distances (up to 108 km; Harwell and Orth 2002) before their negatively buoyant seeds dehisce from the shoot and germinate (Harwell and Orth 2002; McMahon et al. 2014).

There are few studies that have investigated *Z*. *muelleri* germination rates despite it being a prolific species on the east coast of Australia and in New Zealand (Mills and Berkenbusch 2009; Lee et al. 2016; Stafford‐Bell, Chariton, and Robinson 2016; Tan et al. 2023). Successful germination in *Z*. *muelleri* seeds mostly encompass low temperatures (< 16°C) and low salinities (0–16 ppt) (Conacher et al. 1994; Brenchley and Probert 1998; Stafford‐Bell, Chariton, and Robinson 2016). However, germination is likely affected by lesser‐known cues as *Z*. *muelleri*, and similar species can be affected by factors such as anoxia, sediment microbes and burial depth (Conacher et al. 1994; Stafford‐Bell, Chariton, and Robinson 2016; Cumming et al. 2017; Tarquinio et al. 2019). Treatments with high salinities (32 ppt), 24‐h light cycles and temperatures above 16°C see little to no germination in temperate *Zostera* sp. despite these being conditions that would be commonly seen in east Australian estuaries (Stafford‐Bell, Chariton, and Robinson 2016).

Optimal *Z*. *muelleri* germination conditions in the laboratory do not reflect conditions experienced in Australian estuaries, which are highly variable in morphologies and subsequently the environmental parameters within them. Consequently, estuaries are not subject to static salinity and temperature regimes, but rather a wide range of both parameters due to tidal movements, rain events and evaporation (Scanes, Scanes, and Ross 2020). On the east coast of Australia estuarine salinity rests between 25 and 35 ppt, however, it is possible for systems to experience salinities ranging between 0 and 35 ppt during large rain events particularly near river entrances, albeit for a short duration (Eyre and Ferguson 2006; Stafford‐Bell, Chariton, and Robinson 2016). Based on successful laboratory germination, periods of low salinities can encourage successful *Z. muelleri* germination (Stafford‐Bell, Chariton, and Robinson 2016). However, with rainfall on the east coast of Australia decreasing due to climate change (Head et al. 2014), the likelihood of low salinity events inducing germination is decreasing. Considering the importance of sexual reproduction and large flowering events in population resilience (Stafford‐Bell, Chariton, and Robinson 2015; Smith et al. 2016) and the strong influence temperature and salinity have on germination (Stafford‐Bell, Chariton, and Robinson 2016; Smith et al. 2016; Cumming et al. 2017; Tol et al. 2017), acclimation or adaption to warmer temperatures predicted to occur due to climate change will be critical for the survival of *Z. muelleri* in Australia (York et al. 2013; Collier and Waycott 2014).

There are multiple mechanisms that may influence the capacity for acclimation or adaption to warmer temperatures. Thermal conditions during various developmental stages can drive natural selection, affect phenotypic plasticity and influence epigenetic changes within the species (Nguyen et al. 2020). Epigenetic variation or changes in the phenotype can often be attributed to thermal priming; the concept that organisms can be ‘hardened’ against subsequent severe temperature stressors by first being exposed to more moderate ones (Nguyen et al. 2020; Pazzaglia et al. 2022). While Z. *muelleri* flowering is heavily temperature reliant (Lekammudiyanse et al. 2024), there have been limited studies on the capacity for thermal priming or how increased temperatures experienced by the parent plant may affect seed performance. Epigenetic modifications have been identified through changes in DNA methylation (a process in which gene expression is altered without changing the underlying DNA) in Z. *muelleri* and another southern hemisphere species, *Posidonia australis*, with primed plants experiencing differences in gene expression coinciding with increases in photosynthetic efficiency (Nguyen et al. 2020). Primed plants also displayed morphological changes with leaf elongation and biomass experiencing a significant reduction during an initial heatwave compared to a subsequent heatwave, demonstrating a ‘stress memory’—where subsequent stressors are alleviated because the organism has previously experienced them (Nguyen et al. 2020). There is also evidence of thermal priming increasing heat tolerance in the seedlings of *Posidonia oceanica*, a mediterranean species (Pazzaglia et al. 2022). While these examples provide evidence on how seagrasses or reproductive mechanisms such as flowering might be affected by elevated temperatures, there is little evidence for marine macrophytes on effects experienced by seeds when maternal plants are exposed to elevated temperatures. In terrestrial plants, transgenerational effects, the idea that traits can be passed down through generations based on parental experiences, are well‐studied and in many cases maternal plants that experience stressful conditions display increased germination and stressor‐specific adaptions (i.e., a drought stressor results in drought adapted offspring) compared to those from maternal plants held in beneficial conditions (Sultan 1996; Sultan, Barton, and Wilczek 2009; Walter et al. 2016).

This study used two thermal plumes generated by coal‐fired power stations as a proxy for a future climate change scenario. Thermal plumes have been studied globally (Robinson 2010; Steinbeck, Schiel, and Foster 2005; Garthwin, Poore, and Vergés 2014) and can provide an avenue to explore in situ effects from predicted warming scenarios. Due to the significance of temperature in both germination and seagrass growth, it is expected that seeds sourced from thermally affected areas will experience differences in germination depending on temperature treatment. As such, the study aimed to investigate whether seeds sourced from plants that grow in elevated temperatures during their development respond differently to temperature treatments than those growing in ambient locations in the thermally affected and a control estuaries.

## 2 Methods

### 2.1 Seed Collection

**FIGURE 1:**
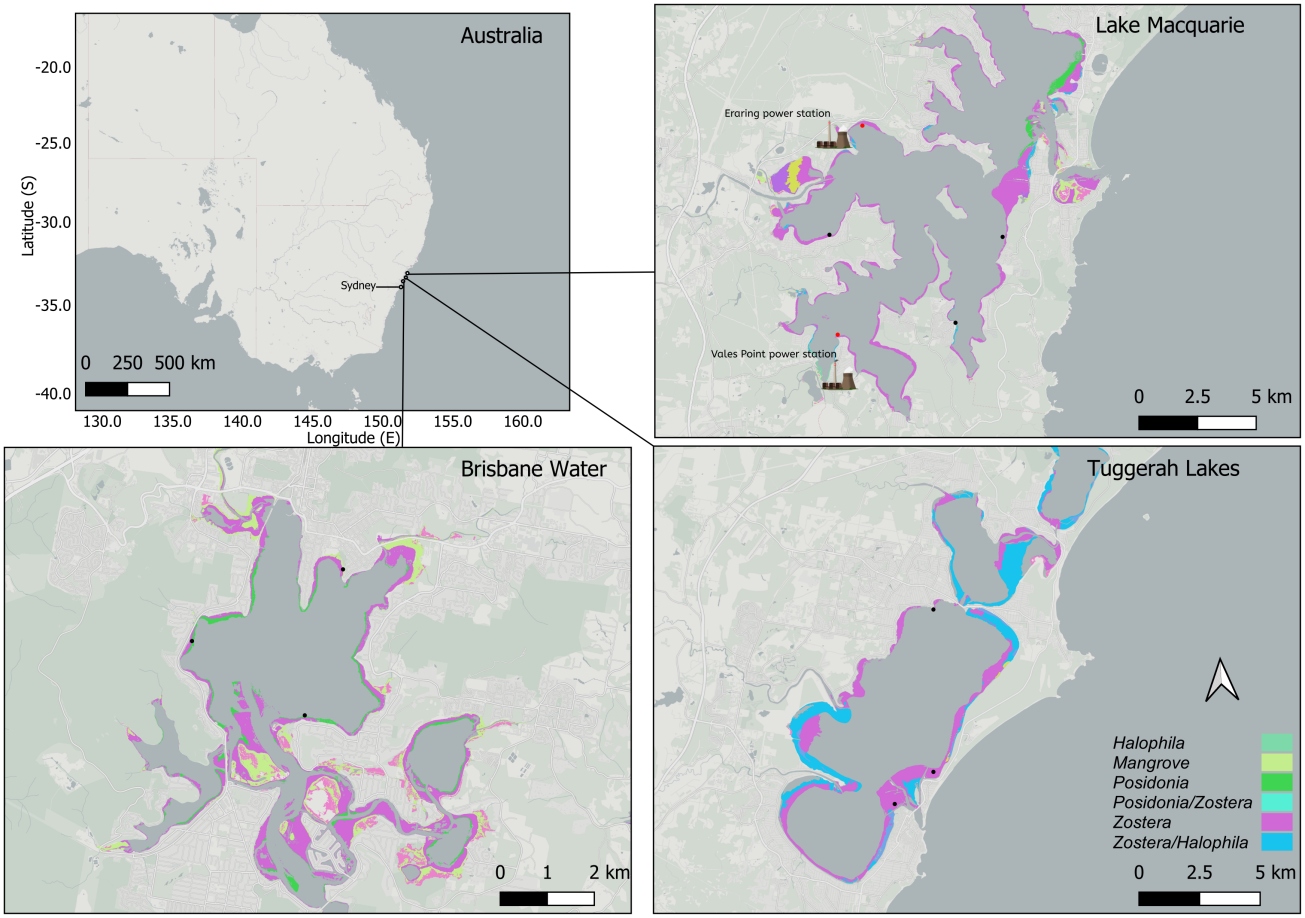
Site map showing where reproductive shoots were collected. In Lake Macquarie, there is two thermally affected locations, which are illustrated by red markers and a power station image. Seagrass mapping data sourced from NSW Department of Primary Industries.

The study ran over 2 years and encompassed two separate flowering seasons. For the first year of study, reproductive shoots were collected from five locations within Lake Macquarie, a barrier estuary on the central coast of NSW, Australia (Figure 1). The perimeter of the lake is dominated by seagrass, most notably *Z. muelleri*. There are two power stations that use the lake for cooling water and discharge the thermal effluent back into the lake at Myuna Bay (Eraring Power Station) and Mannering Park (Vales Point Power Station), resulting in temperatures approximately 2°C–3°C higher than ambient temperatures (Macreadie and Hardy 2018; Suzzi et al. 2023). All sites for this study are within the southern half of the lake to avoid a heavy metal gradient (Roach 2005) and extensive urbanisation in the north of the lake. In the second year of the study, two control estuaries situated near Lake Macquarie were included in the study, Brisbane Water and Tuggerah Lakes. At each of these estuaries, reproductive shoots were collected at three locations.

Reproductive shoots were collected over two different reproductive seasons (2021/2022 and 2023/2024) during peak reproductive effort in this area which falls between November and December (Lekammudiyanse et al. 2024). At thermally affected sites (Myuna Bay and Mannering Park in Lake Macquarie), reproductive shoots were collected late October, while at ambient locations, reproductive shoots were collected late November. The difference in collection times was due to the likelihood of temperature affecting seed maturation times (Smith et al. unpublished). All shoots collected contained mature, dark brown seeds. Reproductive shoots were transported to the laboratory in a 30‐L tub filled with seawater from the collection site. Shoots were then placed outdoors in a shaded area in aerated 50‐L containers with natural seawater (34 ppt) sourced from Swansea Channel, the entrance to Lake Macquarie. After maturation (~4 weeks after collection), negatively buoyant seeds were siphoned from the bottom of the tanks and stored in autoclaved seawater (sourced from Swansea Channel) at 4°C for a maximum of 2 days before being set up in an experiment. To simulate a freshwater pulse and induce germination, seeds were moved from storage, placed into a 30‐mL sample jar of distilled water, stirred lightly for 5 min and then stored for 24 h to assist in initiating germination (Stafford‐Bell, Chariton, and Robinson 2016; Cumming et al. 2017).

### 2.2 Viability

To determine viability, seeds (*n* = 100) from each site except Gwandalan 21/22 (*n* = 50) were subject to a cut test prior to being placed into the germination experiment (Smith et al. 2016). The cut test is an efficient (both time and economically) test, which involves slicing the seed in half and deems seeds, which are firm and have a blue tinge to be viable (Borza, Westerman, and Liebman 2007; Smith et al. 2016) highlighted no difference between tetrazolium staining and a cut test in determining seed viability. However, to ensure the cut test was being completed correctly, tetrazolium staining was also performed on four sites (Myuna Bay, Mannering Park, Murrays Beach and Yarrawonga Park; *n* = 100) and compared by ANOVA, which found no significant differences in viability between the two tests (*p* = 0.396, *F* = 0.728, DF = 1). Consequently, in the second year, only the cut test was performed. Unfortunately, in Brisbane Water and Tuggerah Lakes tanks, aerator failures resulted in usable seeds from only one site from each estuary, which were Saratoga and Pipeclay Point, respectively. This meant that the original design of *n* = 3 control sites in two separate control estuaries was not accomplished, and because of no significant differences between estuaries, ambient sites from Tuggerah Lakes and Brisbane Water, were pooled with ambient sites from Lake Macquarie.

### 2.3 Germination Experiment

For each site, 100 seeds were randomly allocated to five petri dishes (*n* = 20 per Petri dish). However, insufficient seeds were collected at Gwandalan in 2021, so three replicates (*n* = 14) were placed in the 16°C and 20°C treatments only. Likewise, Saratoga Point only had sufficient seeds for five replicates (*n* = 20) in the 16°C treatment. Each Petri dish was filled with approximately 5–10 mL of sterilised seawater sourced from the entrance of the lake on a single sampling occasion (34 ppt) and fitted with an autoclaved sponge to reduce evaporation and wet‐strengthened filter paper to ensure the seeds did not move for the duration of the experiment (Tol et al. 2017). Seeds were then placed into plastic containers blacked out with aluminium foil and randomly assigned to one of four (16°C, 20°C, 24°C, 28°C) temperature control cabinets, which allowed no ambient light. Excess seeds from five of the sites were used in an additional five replicates (*n* = 20) to be held at 8 ppt salinity in the 16°C and 28°C treatment to investigate any effect of salinity. In the 16°C treatment, this included all sites except for Gwandalan and Saratoga and in the 28°C treatment it included Mannering Park, Myuna Bay and Pipeclay Point. During the first year, the experiment ran for 136 days and in the second 145 days, seeds were checked for germination every 2–3 days and evaporative loss topped up when required with saline solution (Cumming et al. 2017). Water was siphoned and changed fortnightly or earlier in the case of unusual evaporation to ensure salinity did not fluctuate. Temperature treatments and salinity were based on likely scenarios within the estuaries where the seeds were sourced. On conclusion of the first year of experiment, seeds were subjected to another freshwater pulse for 48 h and results recorded for separate analysis.

### 2.4 Statistical Analysis

To determine the effect of seed location (Lake Macquarie—ambient and thermally affected; Brisbane Water—ambient, Tuggerah Lakes—ambient) on mean time to germination (MTG), cox models were fit to seed germination data using the R package ‘survival’ (Therneau 2022) and significant differences identified using estimated marginal means with the ‘emmeans’ package with a Tukey adjustment (McNair, Sunkara, and Frobish 2012; Tol et al. 2017; Lenth 2022). Because there were no differences detected between ambient treatments regardless of estuary, all seeds were grouped into either ‘ambient’ or ‘thermally affected’ treatments. Non‐proportional hazards were checked using Kaplan–Meier survivorship functions and the model with the lowest Akaike information criterion (AIC) was used (McNair, Sunkara, and Frobish 2012). Germination analyses did not include the freshwater pulse in the first year, which concluded the experiment to avoid inflating the original germination rate.

To determine the influence of temperature treatments and location of seeds on seed germination, a multiple logistic regression model was fit to individual seed data. To ensure the model fit the data and to determine which covariates or interactions (*Site*, *Location*, *Treatment*) significantly influenced the model, deviance tables were constructed on various models and compared using ANOVA with a chi square test. Multicollinearity was tested by calculating the variance inflation factors (Heiberger, Heiberger, and Burt Holland 2015; Cumming et al. 2017). Factors that did not significantly influence the model or suffered from multicollinearity were identified and removed from the model (Site and the interaction of Location and Treatment). As in the cox model, pairwise differences were identified using estimated marginal means with the ‘emmeans’ package with no adjustment (Lenth 2022). The MASS package (Venables and Ripley 2002) was then used to calculate *e* ^β^ and 95% confidence intervals to quantify the influence of location on seed germination.

To compare means of dead seeds on experiment completion and maximum mean germinations pre and pulse post and any interaction between location and pulse, data were first analysed for homogeneity of variance, which was confirmed by plotting residual values vs. fitted values and normality using a Q–Q plot. ANOVA was then used with a Tukey's HSD post hoc to determine where significant differences occurred (Zar 2005).

## 3 Results

### 3.1 Germination and Influence of Temperature Treatments

Overall, the experiment found low germination rates across all temperature treatments with ~2% (*n* = 44) of seeds germinating from ambient locations and ~4% (*n* = 73) of seeds germinating from thermally affected locations despite viability from all locations being > 95%. There were significant differences in MTG, germination likelihood and the number of dead seeds depending on if seeds were sourced from thermally affected sites or if they were from ambient sites.

### 3.2 Mean Time to Germination

**FIGURE 2:**
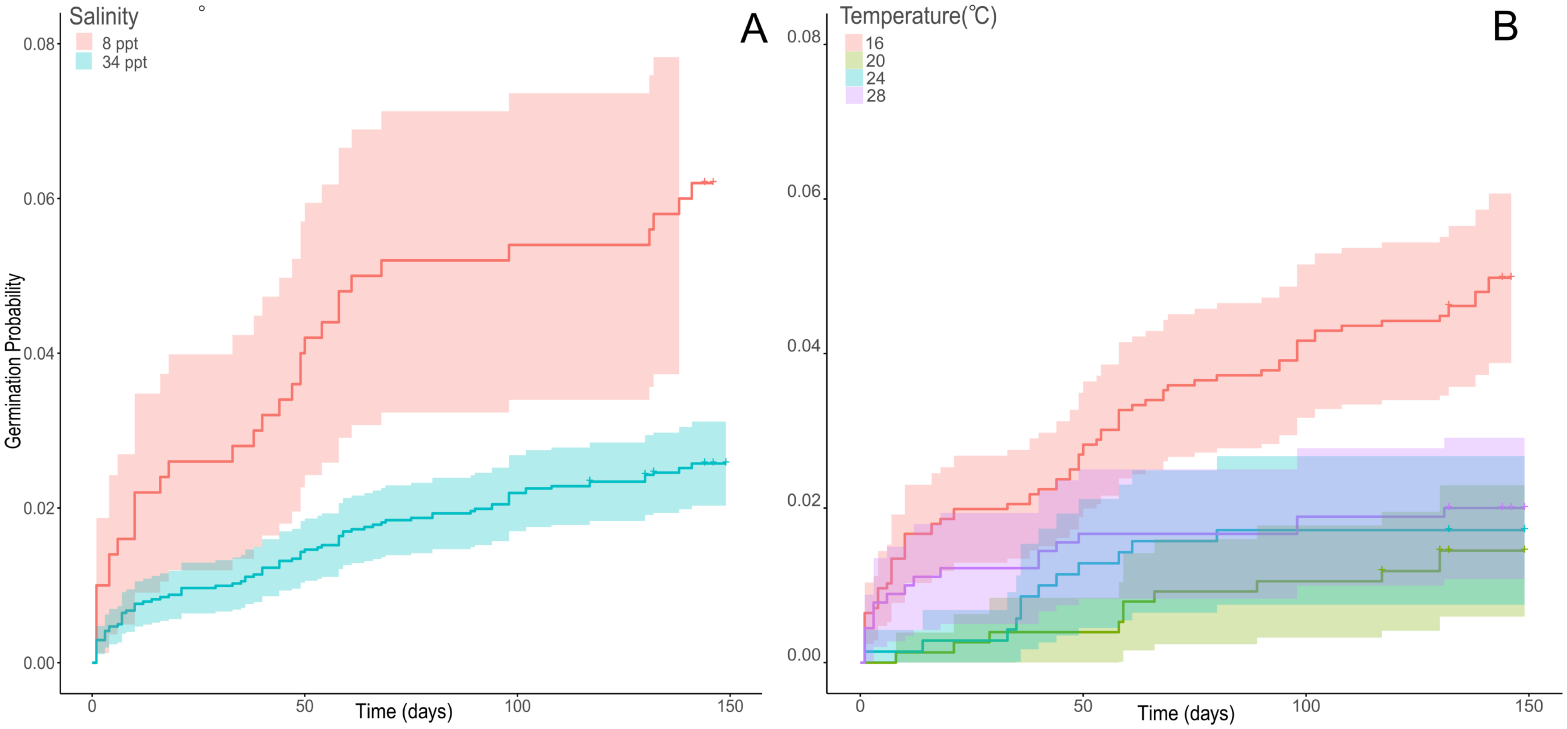
Kaplan–Meier survivorship functions of germination probability based on days of experiment. Panel (A; left) compares germination probabilities of *Zostera muelleri* seeds in two different salinity treatments while panel (B; right) investigates four different temperature treatments.

**FIGURE 3:**
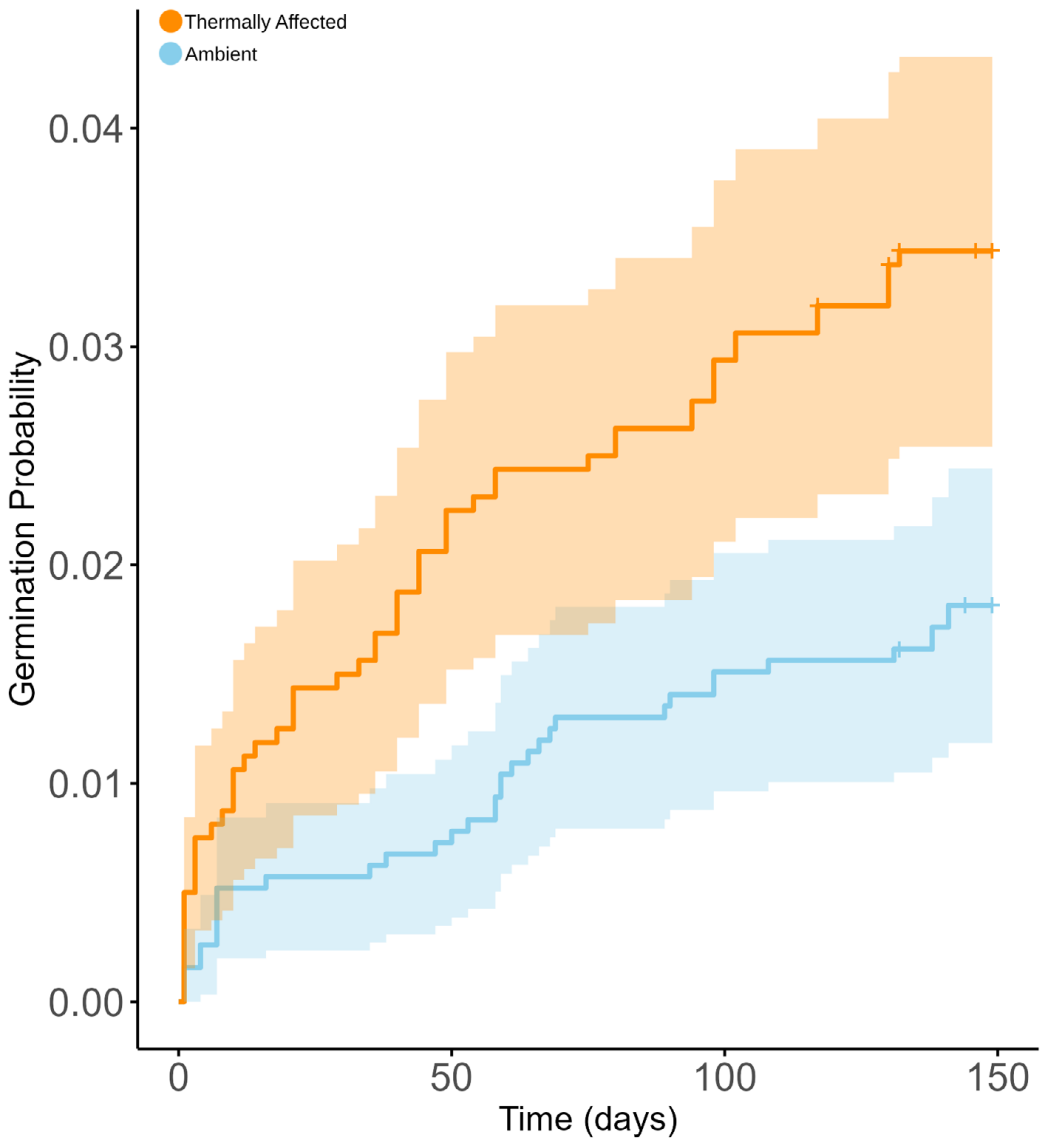
Kaplan–Meier survivorship functions of germination probability based on days of experiment, comparing *Zostera muelleri* seeds from thermally affected and ambient locations. Shading represents a 95% Confidence Interval.

There was no difference in MTG between the two different sampling years. Cox models identified that the 16°C treatment germinated significantly faster than all other treatments (Figure 2; Table 1; *p* < 0.001). Seeds sourced from plants living in thermally affected sites germinated faster (46.4 ± 5.7 days) compared to seeds from ambient locations (52.7 ± 7.3 days) (*p ≤* 0.001; Table 1, Figure 3). Further, seeds in salinities of 8 ppt germinated faster than those in 32 ppt; however, there were no interactions between salinity and location or salinity and treatment.

**TABLE 1:** Mean time to germination results for cox model time to survival analysis paired with emmeans post hoc.

Parameter	Intercept	SE	*Z* ratio	*p*
Thermally affected versus Ambient	0.810	0.192	4.209	**< 0.001**
Salinity	−0.018	0.009	−2.012	**0.044**
16°C–20°C	1.185	0.337	3.328	**< 0.001**
16°C–24°C	1.047	0.364	2.879	**0.0012**
16°C–28°C	1.048	0.348	3.008	**0.004**
20°C–24°C	−0.041	0.450	−0.091	0.928
20°C–28°C	−0.040	0.438	−0.093	0.925
24°C–28°C	0.001	0.459	0.000	0.999

*Note:* Bolded values are significant (*p* < 0.05).

### 3.3 Effect of Temperature and Salinity on Maximum Germination

**FIGURE 4:**
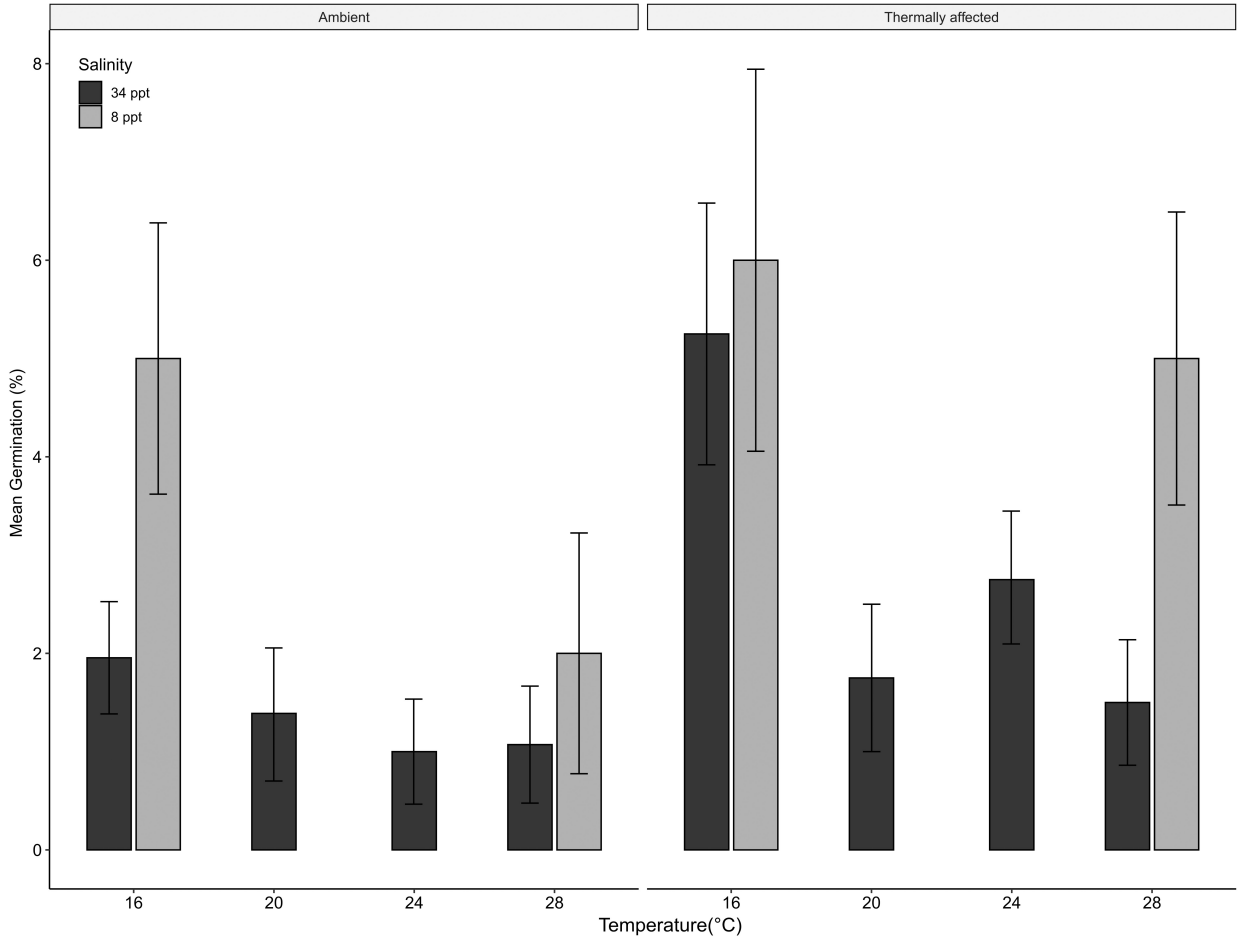
Mean germination (%) from seeds in two different salinity levels at ambient (left panel) and thermally affected sites (right panel) averaged over both sampling years.

Mean germination was affected by both location and temperature treatment; however, there was no interaction between the two covariates, highlighting that thermally affected seeds did not respond differently to temperature treatments. Salinity increased germination (*p* = 0.035, Table 2) but did not have any significant interaction effects between treatment and source. Emmeans post hoc highlighted that all treatments had significantly less germination when compared to the 16°C treatment but were not different from each other (Figure 4; Table 2). Further, there were significant differences between the 16°C treatments held at 32 ppt salinity in thermally affected locations compared with the 16°C in ambient locations, with germination rates of 5.5% (±1.08) and 2.8 (±0.58), respectively (Figure 4).

**TABLE 2:** Multiple binomial logistic regression performed on individual seed data highlighting germination probability for *Zostera muelleri* seeds sourced from ambient locations and thermally affected locations, subjected to four different temperature treatments (16°C, 20°C, 24°C, 28°C).

Parameter	Estimate	SE	*Z* ratio	*p*
Thermally affected—control	0.894	0.227	−3.935	**< 0.001**
salinity	−0.020	0.009	−2.103	**0.035**
16°C–20°C	1.244	0.341	3.584	**< 0.001**
16°C–24°C	1.088	0.331	3.283	**< 0.001**
16°C–28°C	0.824	0.283	2.914	**< 0.001**
20°C–24°C	−0.136	0.421	−0.322	0.747
20°C–28°C	−0.400	0.387	−1.034	0.301
24°C–28°C	−0.264	0.377	−0.700	0.484

*Note:* Bolded values are significant.

Binomial regression (Table 2) highlighted seeds sourced from thermally affected sites were 145% more likely to germinate compared to seeds sourced from ambient locations when holding temperature constant (95% CI [0.449, 1.339]).

### 3.4 Seed Mortality

**FIGURE 5:**
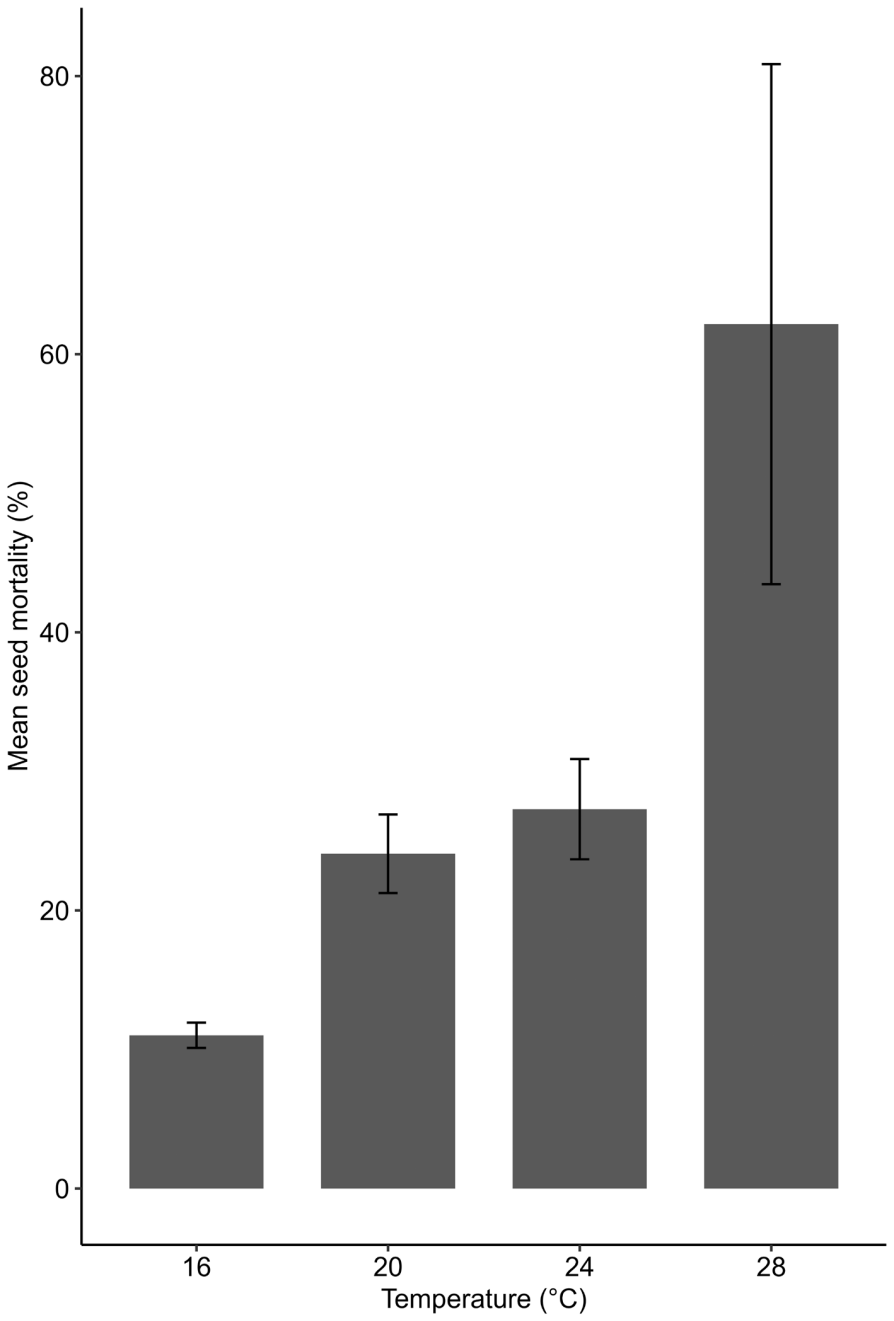
Mean seed mortality (±SE) across all treatments and both years.

There were no differences between thermally affected or control location seed mortality, nor were there any interactions to suggest that thermally affected seeds had differing rates of mortalities at different temperatures compared with control locations. Seeds experienced lower mortalities at lower temperatures in both thermally affected and ambient locations, with seed mortality being significantly lower in the 16°C treatment compared to the 28°C treatments (11.02% ± 0.91% and 62.16% ± 18.69%, respectively; Figure 5; ANOVA; *p* < 0.001).

### 3.5 Influence of a Freshwater Pulse

**FIGURE 6:**
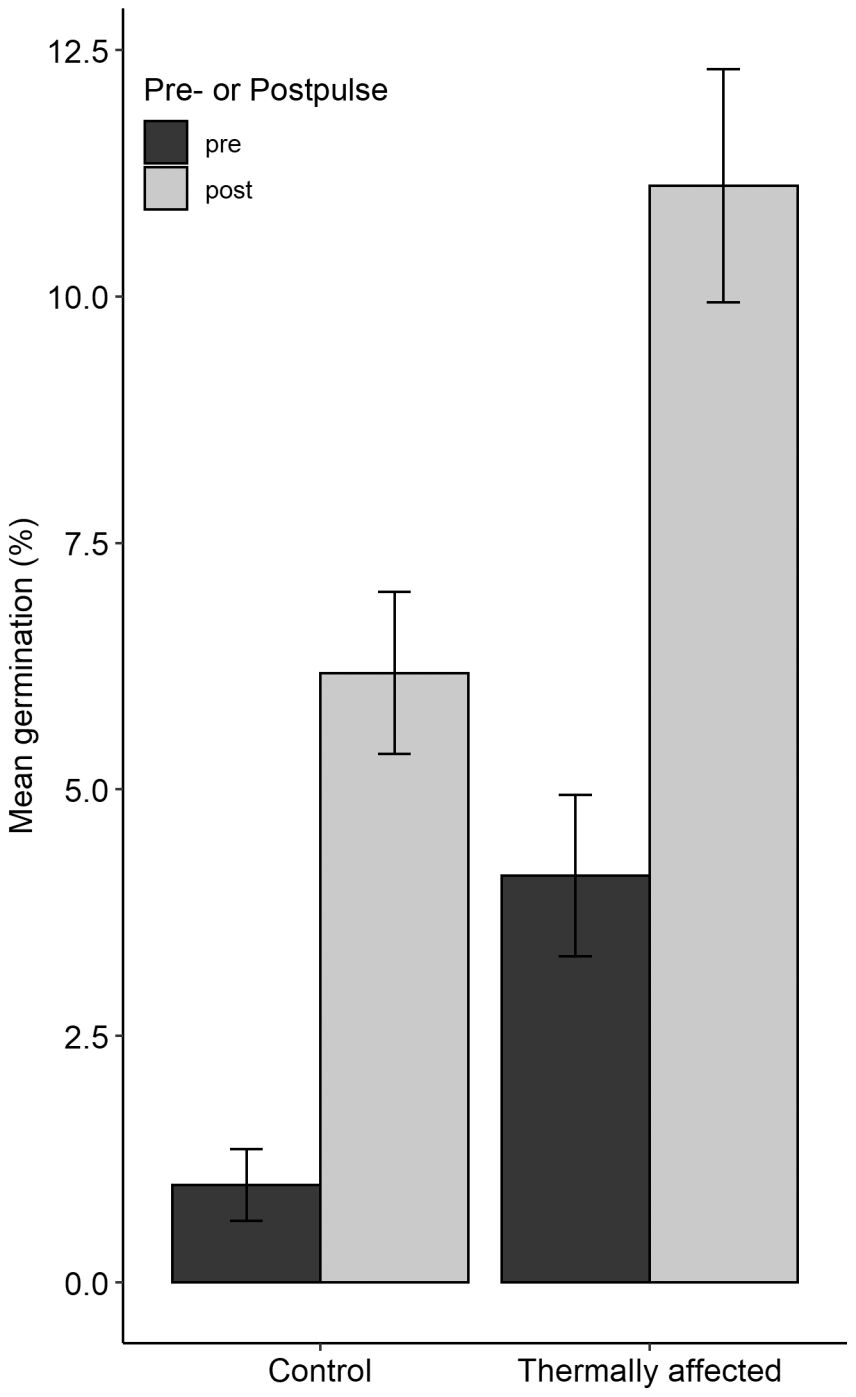
Comparison of mean (±SE) germination (%) pre (black bars) and post (grey bars) a 48 h freshwater pulse on seeds from the first year of sampling.

After 146 days of experimentation, a freshwater pulse was conducted on conclusion of the first year of the experiment to determine whether thermally affected sites and control locations would respond differently to a freshwater pulse after being subject to different temperature treatments. Overall, the pulse significantly increased germination in control and thermally affected seeds by 400% and 180%, respectively (ANOVA; *p* < 0.001, Figure 6). There were no significant interaction effects between pulse and seed location and thus the pulse was not repeated for the second year.

## 4 Discussion

This experiment provides evidence that *Zostera muelleri* seeds from thermally affected areas have higher germination rates than those from ambient locations. Counter‐intuitively, this suggests that, to some degree, higher temperatures projected with climate change may increase *Z. muelleri* germination success in estuarine environments. However, this effect is likely counteracted by the fact that suitable conditions for germination will become less prevalent as global temperatures increase.

The differences in germination between thermally affected and control locations suggests that *Z*. *muelleri* seeds sourced from thermally affected areas may be higher quality seeds than those from ambient locations. The exact mechanisms behind this are unknown, and we did not notice any obvious differences in seed size between the two treatments, which may be one indicator of this (Smith et al. 2022). There is evidence from the terrestrial environment suggesting that increased seed quality and germination can be a form of transgenerational plasticity. In terrestrial environments, while species specific (Germain and Gilbert 2014), there are examples of maternal plants that are subjected to stressful conditions experiencing higher germination rates than those subject to suitable conditions (Germain, Caruso, and Maherali 2013; Walter et al. 2016). This response is theorised to reduce exposure to unfavourable conditions during sensitive early life cycle stages (Walter et al. 2016). Potentially, higher temperatures experienced in the thermally affected sites are facilitating some form of transgenerational plasticity in *Z. muelleri* like what is seen in terrestrial examples. However, thermally affected seeds did not show any differences from ambient locations in germination at higher temperatures, demonstrating an inability for any potential transgenerational effects to inhibit the impact higher temperatures have on reducing germination. Due to the severity of the elevated temperatures in thermally affected areas, which maternal plants experience (~2°C–3°C average), this may demonstrate a plasticity ceiling for the temperate *Z. muelleri* in terms of any germination increases. While most *Zostera* sp. have shown low germination rates at combinations of high temperatures and high salinities, there are exceptions. Tropical *Z*. *muelleri* appears to germinate more readily and not be inhibited as severely by high temperatures and high salinity (34 ppt), with higher germination rates of 20%–36% depending on temperature (Tol et al. 2017). Likewise, *Zostera marina* saw increased germination at higher salinities with higher temperatures (Xu et al. 2016). Other transgenerational adaptions to increased temperatures have been observed in *Z. marina*, which highlighted that exposure to a heat wave by a parent plant stimulated an increase in above‐ground biomass and the favouring of offspring shoot production rather than parent shoot maintenance (DuBois, Williams, and Stachowicz 2020). Similarly, population specific resilience has been identified in another seagrass species (*Halophila ovalis*) for both abiotic (salinity fluctuations; Webster et al. 2021) and biotic factors (grazing; O'Dea et al. 2022).

Increasing germination from thermally stressed plants may be a way for *Z*. *muelleri* to maintain population resilience when faced with a warming climate. Seeds having a higher likelihood to germinate would allow seagrass species to germinate more frequently and in the absence of a storm surge (which would bring favourable temperatures + salinities), which is important for species recovery after a stress event (Smith et al. 2016). An increase in germination from thermally stressed plants could lead to greater population resilience by increasing the window of ‘suitable conditions’ for germination, which typically are shown to be cooler temperatures and lower salinities (Conacher et al. 1994; Stafford‐Bell, Chariton, and Robinson 2016); conditions usually limited during winter months where storms are more frequent and temperatures cooler.

This study also reinforced that *Z*. *muelleri* is likely dependent on freshwater pulses and concomitant cooler waters, which come with storm surges to significantly increase germination rates. This is similar to other species where lower temperatures and salinities saw much higher germination rates (Stafford‐Bell, Chariton, and Robinson 2016; Cumming et al. 2017). Seeds in this study experienced increased germination and faster MTG at lower salinities. In a different species, *Zostera nigricaulis*, the effect of a freshwater pulse to begin the experiment eliminated the influence of salinity on seed germination (Cumming et al. 2017). This would suggest that in our study, which also had a freshwater pulse to begin the experiment, salinity should not have influenced germination. Possibly, the much larger difference in salinities between the 8 ppt treatment and the 34 ppt treatment of the present study explains why salinity still had an effect on our seeds, as Cumming et al. (2017) examined a much tighter salinity range of 25–35 ppt.

Overall, the experiment saw very low germination rates; however, this is not entirely uncommon for temperate *Z*. *muelleri* subject to salinity treatments of > 32 ppt. It is also important to consider that large amounts of seeds are being produced, which means low germination still results in a high number of seedlings. This is demonstrated by a closely related species, *Z. marina*, where in situ experiments have seen similar germination rates of 4.7%–13.8% (*n* = 50,000; Orth et al. 2003). Typically, higher germination rates are seen in low salinity treatments (< 20 ppt) and treatments of 30+ ppt rarely exceed 0%–10% germination in temperate *Z*. *muelleri* (see Conacher et al. 1994; Stafford‐Bell, Chariton, and Robinson 2016). Due to these low numbers, it is likely that some other germination cue such as dissolved oxygen content (Brenchley and Probert 1998) or the effect of sediment microbes is required (Tarquinio et al. 2019). While more suitable conditions may be uncommon in estuaries, they are not entirely unlikely in a near‐shore environment where *Z*. *muelleri* resides which would receive high amounts of freshwater from runoff (York et al. 2013; Collier et al. 2014).

Despite low germination numbers, the outcomes from this paper highlight significant ecological implications and may mean that temperate *Z*. *muelleri* is approaching an optimum period of increased germination rates as temperatures trend upwards, given we recorded higher germination rates in seeds sourced from adult plants living in higher temperatures. The evidence presented here suggests that under certain scenarios, *Z*. *muelleri* will experience a net increase in germination as temperatures increase. However, this would depend on other reproductive metrics such as reproductive shoot densities, spathe counts and seed viabilities remaining static under increasing temperatures. It would also be advantageous to understand if this interaction is isolated to estuaries with thermal plumes or if systems which experience similar ambient temperatures to the plumes also show a similar germination response.

## Author Contributions

**Tom Moir:** conceptualization (equal), data curation (lead), formal analysis (lead), funding acquisition (lead), investigation (lead), methodology (equal), writing – original draft (lead), writing – review and editing (equal). **Megan J. Huggett:** conceptualization (supporting), funding acquisition (equal), methodology (supporting), supervision (equal), writing – review and editing (equal). **Timothy M. Smith:** conceptualization (equal), methodology (equal), supervision (supporting), writing – review and editing (equal). **Troy F. Gaston:** conceptualization (equal), formal analysis (supporting), funding acquisition (equal), methodology (equal), supervision (equal), writing – review and editing (equal).

## Conflicts of Interest

The authors declare no conflicts of interest.

## Data Availability

All data analysed in this paper are publicly available at the following reference: Tom Moir. (2024). Thermal priming boosts germination in *Z. muelleri* [Data set]. Kaggle. https://doi.org/10.34740/KAGGLE/DSV/8330314.
